# Distinct evolutionary histories of the DNA-A and DNA-B components of bipartite begomoviruses

**DOI:** 10.1186/1471-2148-10-97

**Published:** 2010-04-08

**Authors:** Rob W Briddon, Basavaprabhu L Patil, Basavaraj Bagewadi, Muhammad Shah Nawaz-ul-Rehman, Claude M Fauquet

**Affiliations:** 1 National Institute for Biotechnology and Genetic Engineering (NIBGE), Faisalabad, Pakistan; 2 ILTAB, Donald Danforth Plant Science Center, 975 North Warson Road, St Louis, MO, USA; 3 Department of Plant Pathology, University of Kentucky, Lexington, KY, USA

## Abstract

**Background:**

Viruses of the genus *Begomovirus *(family *Geminiviridae*) have genomes consisting of either one or two genomic components. The component of bipartite begomoviruses known as DNA-A is homologous to the genomes of all geminiviruses and encodes proteins required for replication, control of gene expression, overcoming host defenses, encapsidation and insect transmission. The second component, referred to as DNA-B, encodes two proteins with functions in intra- and intercellular movement in host plants. The origin of the DNA-B component remains unclear. The study described here was initiated to investigate the relationship between the DNA-A and DNA-B components of bipartite begomoviruses with a view to unraveling their evolutionary histories and providing information on the possible origin of the DNA-B component.

**Results:**

Comparative phylogenetic and exhaustive pairwise sequence comparison of all DNA-A and DNA-B components of begomoviruses demonstrates that the two molecules have very distinct molecular evolutionary histories and likely are under very different evolutionary pressures. The analysis highlights that component exchange has played a far greater role in diversification of begomoviruses than previously suspected, although there are distinct differences in the apparent ability of different groups of viruses to utilize this "sexual" mechanism of genetic exchange. Additionally we explore the hypothesis that DNA-B originated as a satellite that was captured by the monopartite progenitor of all extant bipartite begomoviruses and subsequently evolved to become the integral (essential) genome component that we recognize today. The situation with present-day satellites associated with begomoviruses provides some clues to the processes and selection pressures that may have led to the "domestication" of a wild progenitor of the DNA-B component.

**Conclusions:**

The analysis has highlighted the greater genetic variation of DNA-B components, in comparison to the DNA-A components, and that component exchange is more widespread than previously demonstrated and confined to viruses from the Old World. Although the vast majority of New World and some Old World begomoviruses show near perfect co-evolution of the DNA-A and DNA-B components, this is not the case for the majority of Old World viruses. Genetic differences between Old and New World begomoviruses and the cultivation of exotic crops in the Old World are likely factors that have led to this dichotomy.

## Background

The family *Geminiviridae *consists of phytopathogenic viruses with characteristic twinned, quasi-isometric virions encapsidating genomes of circular single-stranded (ss)DNA. Taxonomically the geminiviruses are divided into four genera, three of which (*Mastrevirus*, *Curtovirus *and *Topocuvirus*) consist of viruses with monopartite genomes only. In contrast, the genus *Begomovirus *consists of viruses with either monopartite or bipartite genomes [1]. Prior to 1990 all begomoviruses for which Koch's Postulates had been satisfied using cloned genomes were bipartite. Demonstration of the infectivity of a single component for two begomoviruses causing yellow leaf curl disease of tomato (now known as Tomato yellow leaf curl virus (TYLCV) and Tomato yellow leaf curl Sardinia virus (TYLCSV)) convinced the geminivirus community of the fact that begomoviruses with a single genomic component existed [2,3]. Since then more than 133 begomovirus species having monopartite genomes have been identified and all originate from the Old World (OW). Remarkably, no monopartite begomoviruses native to the New World (NW) have been identified, although recently TYLCV was inadvertently introduced [4].

Within the last few years the vast majority of monopartite begomoviruses have been shown to associate with ssDNA satellites known as betasatellites. Betasatellites are sequence unrelated to their helper begomoviruses and depend on the helper viruses for replication, movement and encapsidation in plants and transmission between plants [5]. In addition, the majority of begomovirus-betasatellite complexes associate with a further class of ssDNA components for which the name alphasatellites has been proposed (formerly referred to as DNA 1; Briddon et al., manuscript in preparation). These are described as satellite-like (due to the fact that they are capable of autonomous replication in plant cells and by definition satellites require a helper virus for replication) and are sequence unrelated to their helper begomoviruses, which they require for movement in plants and transmission between plants [5]. Surprisingly alphasatellites are believed to have originated with another family of ssDNA containing viruses, the nanoviruses [6].

The two components of bipartite begomoviruses are referred to as DNA-A and DNA-B. DNA-A encompasses all virus-encoded functions required for DNA replication, control of gene expression, overcoming host defenses and encapsidation, whereas DNA-B encodes two proteins involved in intra- and intercellular movement [7]. The two components share little sequence identity with the exception of a ~200 nucleotide sequence with typically greater than 85% identity known as the common region (CR). The CR encompasses an absolutely conserved (among geminiviruses) hairpin structure containing, within the loop, the nonanucleotide sequence (TAATATTAC) that marks the origin of virion-strand DNA replication, and repeated sequences (known as "iterons") that are the recognition sequences for binding of the DNA-A-encoded replication-associated protein (Rep: a rolling-circle replication initiator protein that is the only virus-encoded product required for viral DNA replication) [8,9]. The CR thus functions to maintain the integrity of the divided genome, ensuring that replication of both components can be initiated by the DNA-A-encoded Rep [10-12].

Despite having a mechanism to maintain the integrity of their split genomes, component exchange, referred to as pseudo-recombination for begomoviruses, does occur [13-16]. In most cases the mechanism of capture is by a process known as "regulon grafting" wherein the DNA-A component donates its CR, by recombination, to the DNA-B being captured, resulting in a new dependent interaction between two components [17]. Similar origin of replication donation has also been shown to occur for the begomovirus-associated betasatellites although, for reasons that remain unclear, such pseudo-betasatellite molecules are at a selective disadvantage with respect to the parental betasatellite and appear not to be maintained [18].

Although the two components of the majority of bipartite begomoviruses have an obligate relationship, this is not true of all. Both Tomato yellow leaf curl Thailand virus (TYLCTHV) and Sri Lankan cassava mosaic virus(SLCMV) DNA-A components are able to induce symptomatic infections of the experimental host *Nicotiana benthamiana *in the absence of their cognate DNA-Bs [17,19]. These viruses likely represent evolutionary intermediates between monopartite and bipartite begomoviruses. The fact that TYLCTHV occurs in the field associated with either a DNA-B or a betasatellite is consistent with this hypothesis [20,21]. Although all isolates of SLCMV so far characterised are associated with a DNA-B, the potential for this virus to productively interact with a betasatellite has been demonstrated experimentally [17].

Many geminivirus infections are associated with smaller than unit length virus components that are deletion mutants, as reviewed by Patil and Dasguta [22]. These molecules are known as defective interfering (di) DNAs due to their capacity to interfere with virus infection, reducing virus DNA levels and symptom severity [23]. Surprisingly the diDNAs associated with bipartite begomoviruses are derived almost exclusively from the DNA-B component. It is unclear at this time whether this represents a preferential production of diDNAs from DNA-B or that diDNAs are produced equally from both components, but those derived from the DNA-B component are selectively maintained. Sunter *et al*. [24] showed that the super-coiled form of Tomato golden mosaic virus (TGMV) DNA-B is more sensitive to S1 nuclease digestion, indicative of a difference in the structure of the DNA-A and DNA-B components, which might partly explain the differential generation of diDNAs. The accumulation of three times the amount of DNA-B over DNA-A in a typical TGMV infected plant is a factor in the phenomenon [25]. It is possible that DNA-A derived sub-genomic molecules interfere excessively with virus replication and are thus selected against, although there is no evidence to support this hypothesis. There is however an example found in nature, where East African cassava mosaic virus (EACMV) accumulates DNA-A defective molecules and expressing milder symptoms, the accumulation of the diDNA-A is done at the expense of the DNA-B accumulation [26].

The ability of the DNA-A components of bipartite begomoviruses to spread in plants in the absence of DNA-B without inducing symptoms led to the hypothesis that symptoms are a feature of virus movement in plant tissues rather than replication [27]. Consistent with this hypothesis the MP or NSP proteins (but not both for a single species) have been shown to be symptom determinants [27-29].

Mutagenesis studies with the monopartite begomovirus TYLCV have shown that the CP, V2 and C4 proteins mediate nuclear shuttling (CP) and cell-to-cell movement (V2, C4), respectively, the functions carried out by the DNA-B-encoded NSP and MP of bipartite viruses [30]. A major difference, however, was that these were unable to mediate movement through mesophyll and epidermal cells, with the consequence that TYLCV is essentially phloem restricted, in contrast to some bipartite begomoviruses. It is tempting to speculate that the lack of monopartite begomoviruses in the NW is due/related to the absence of the V2 gene.

Using pairwise sequence comparisons and phylogenetic studies we have compared the molecular diversity of the DNA-A and DNA-B components of bipartite begomoviruses. We show that there are distinct differences in the sequence distribution of the DNA-Bs of begomoviruses originating from the Old and New Worlds. Although the viruses from the NW behave in a uniform manner, the begomoviruses from the OW show a group/host specific distribution in pairwise sequence comparisons. The significance of these findings is discussed and the hypothesis that present-day begomovirus DNA-B components originated as satellites is explored.

## Results

### Phylogenetic analysis of geminivirus genome (or DNA-A component) sequences

**Figure 1:**
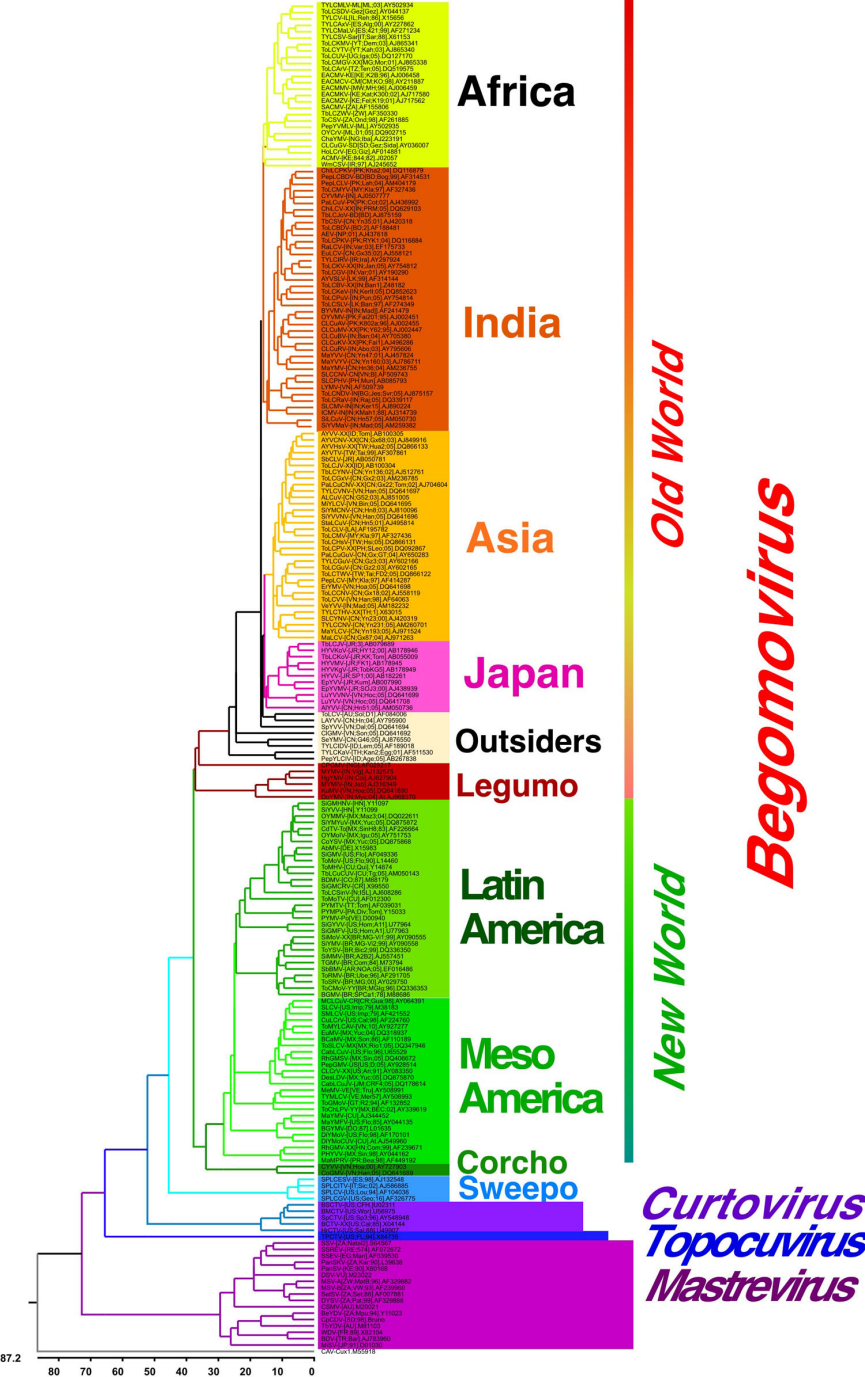
**Phylogenetic tree constructed from an alignment of 212 DNA-A component sequences of geminiviruses**. The sequences used are representative of the 212 geminivirus species recognized as of December 2006. The scale at the basis of the diagram is the pairwise distance expressed as percentage dissimilarity.

A phylogenetic tree constructed from an alignment of the complete genome (or DNA-A component) sequences of 212 geminiviruses (one sequence representing each geminivirus species) is shown in Figure 1. This shows the grouping of begomoviruses according to either geographical origin or the host from which the viruses were isolated, as noted previously [31,32]. The begomoviruses from the OW segregate into clusters originating from Africa, India, Asia, and Japan. However, there is some overlap of the Asian and Indian clusters, likely due to the geographic continuity of these regions and consequent lack of barriers to spread the viruses and their vectors. In addition there are a growing number of viruses that do not fit neatly into these geographic or host based groupings that we shall henceforth refer to as "outsiders". These viruses originate from Indo-China, Indonesia and Australia.

The begomoviruses originating from the NW form a separate cluster from the OW viruses and group according to origin as either from Latin America or Meso America. Closely related to the NW begomoviruses are two species originating from Vietnam isolated from *Corchorus *(hereafter referred to as "Corchovirus") [33,34]. Two further groups of viruses, those infecting a range of legumes originating from India and Southeast Asia (hereafter called "Legumovirus"), and a set of viruses isolated from *Ipomoea *spp. (particularly sweet-potato) originating from America, Asia and Europe (hereafter called "Sweepovirus") are distinct from, and basal to all other begomoviruses. This apparently anomalous position in the phylogenetic tree of these otherwise typical begomoviruses likely reflects a distinct evolutionary history. For the legumoviruses this has been suggested to be due to genetic isolation in their host species [15].

### PASC analysis of geminivirus genome (or DNA-A component) sequences

**Figure 2:**
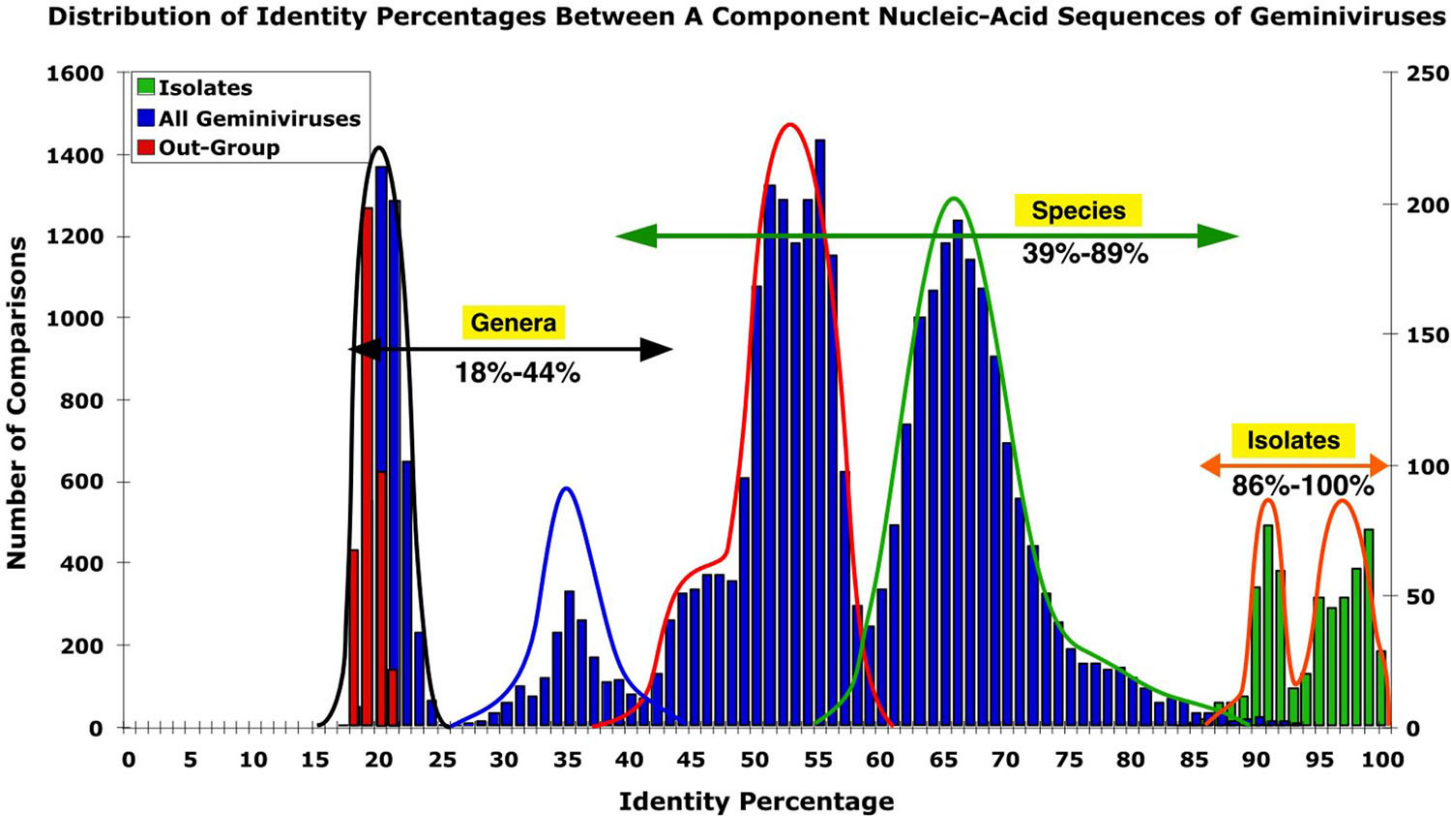
**PASC analysis of 672 sequences of the complete genomes (or DNA-A components) of geminiviruses**. Species comparisons are plotted on the left axis (blue bars) whereas isolate comparisons are plotted separately on the right axis (green bars). The out-group used is the sequence of Chicken anemia virus (#M55918)(red bars).

The molecular diversity of virus sequences is conveniently analysed by pairwise sequence comparison (PASC) [35-39]. As of December 2006, there were 672 complete genome (or DNA-A component) sequences of geminiviruses available in the databases. Based on the presently applicable species demarcation criteria these belong to 212 geminivirus species [37]. A PASC analysis of the sequences reveals a multimodal distribution with six major peaks scattered between 19 and 100% identity (Figure 2).

### Phylogenetic analysis of the DNA-A and DNA-B components of bipartite begomoviruses

**Figure 3:**
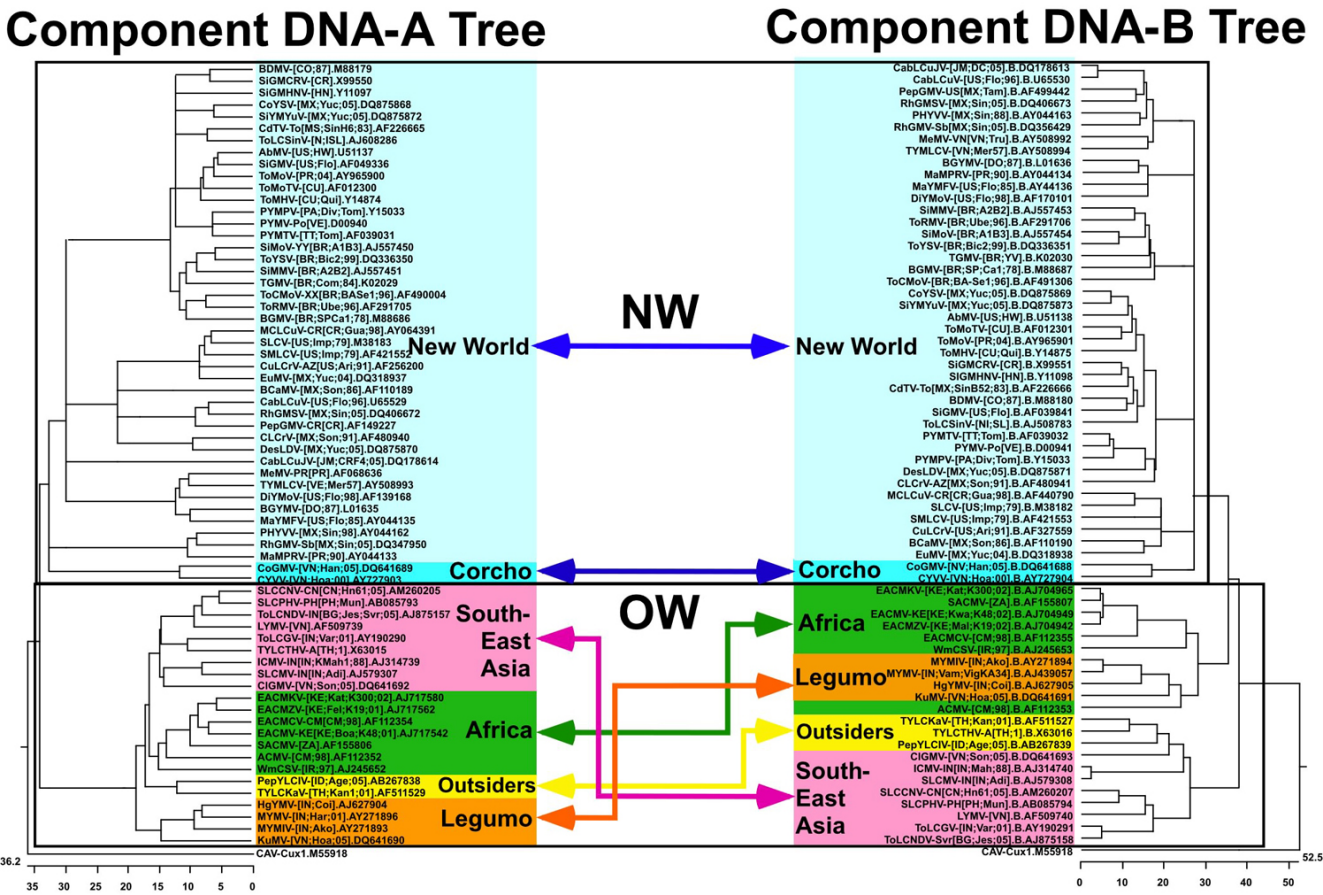
**Phylogenetic trees derived from alignments of the components of bipartite begomoviruses**. The tree based on the DNA-A sequences is shown on the left whereas that derived from the DNA-B sequences is shown on the right. Colours are used to highlight groups of sequences with distinct geographical origins. Branches having bootstrap values lower than 50% have been collapsed. The scale at the base of the diagram is the pairwise distance expressed as percentage dissimilarity.

Of the 672 genome (or DNA-A component) sequences, 182 are of virus isolates of bipartite begomoviruses, belonging to 66 species, for which the cognate DNA-A and DNA-B (isolated from the same plant) sequences are available. Figure 3 is a comparison of the phylogenetic trees resulting from separate multiple alignments of the DNA-A and DNA-B sequences of selected single representatives of each of the 66 species.

The tree based on DNA-A component sequences shows the separation of viruses from the NW (with the corchoviruses) and the OW (Figure 3, left panel. For complete representation of the results summarized in Figure 3, see Panel A, Additional file 1 Figure S1: Phylogenetic trees constructed from an alignment of DNA-A (A) and DNA-B (B) component sequences of 182 bipartite begomoviruses). As in the earlier tree, the OW viruses cluster according to geographic origin or the host from which they were isolated. Two species of the "outsider" group segregate between, and are roughly equidistant to, the OW viruses and the legumoviruses. The two corchoviruses segregate with the NW viruses even though they originate from the OW (Vietnam). The tree based on DNA-B component sequences is similar to that obtained from DNA-A component sequences, and shows the separation of viruses from the NW (with corchoviruses) from the OW viruses (Figure 3, right panel). However, the members of the "outsiders" species that are distinct based on DNA-A sequences cluster with the viruses from Southeast Asia. Significantly, the ACMV DNA-B segregates with, and is basal to, the legumoviruses rather than associating with the DNA-Bs of the other viruses originating from Africa.

A phylogenetic tree based on all DNA-B components of begomoviruses for which a cognate DNA-A is available (182 DNA-B sequences) (see Panel B of Additional file 1 Figure S1: Phylogenetic trees constructed from an alignment of DNA-A (A) and DNA-B (B) component sequences of 182 bipartite begomoviruses) highlights the propensity of these viruses to exchange DNA-B components. For example, most MYMV and MYMIV isolates trans-replicate a similar DNA-B component with the exception of five isolates of MYMV that have a distinct DNA-B, indicative of component exchange between these two species, as noted previously [15,16,40]. Similarly, all isolates of EACMV, EACMZV and SACMV have similar DNA-B components [14]: ToLCGV-[IN:Var:01] (AY190291) shares a DNA-B with ToLCNDV, PuYVMV (AY184488) and SLCCNV-[VN:B]; and SLCMV and ICMV share a DNA-B [17]. Despite the fact that there are over twice the numbers of sequences available for bipartite begomoviruses originating from the NW, few examples [41] of component exchange have been detected in NW viruses. Thus, at least for bipartite begomoviruses originating from the OW, were species demarcation based upon DNA-B sequences, the range of species would look somewhat different than it does under the present classification system entirely based upon DNA-A sequences.

### PASC analysis of DNA-A component sequences of bipartite begomoviruses

**Figure 4:**
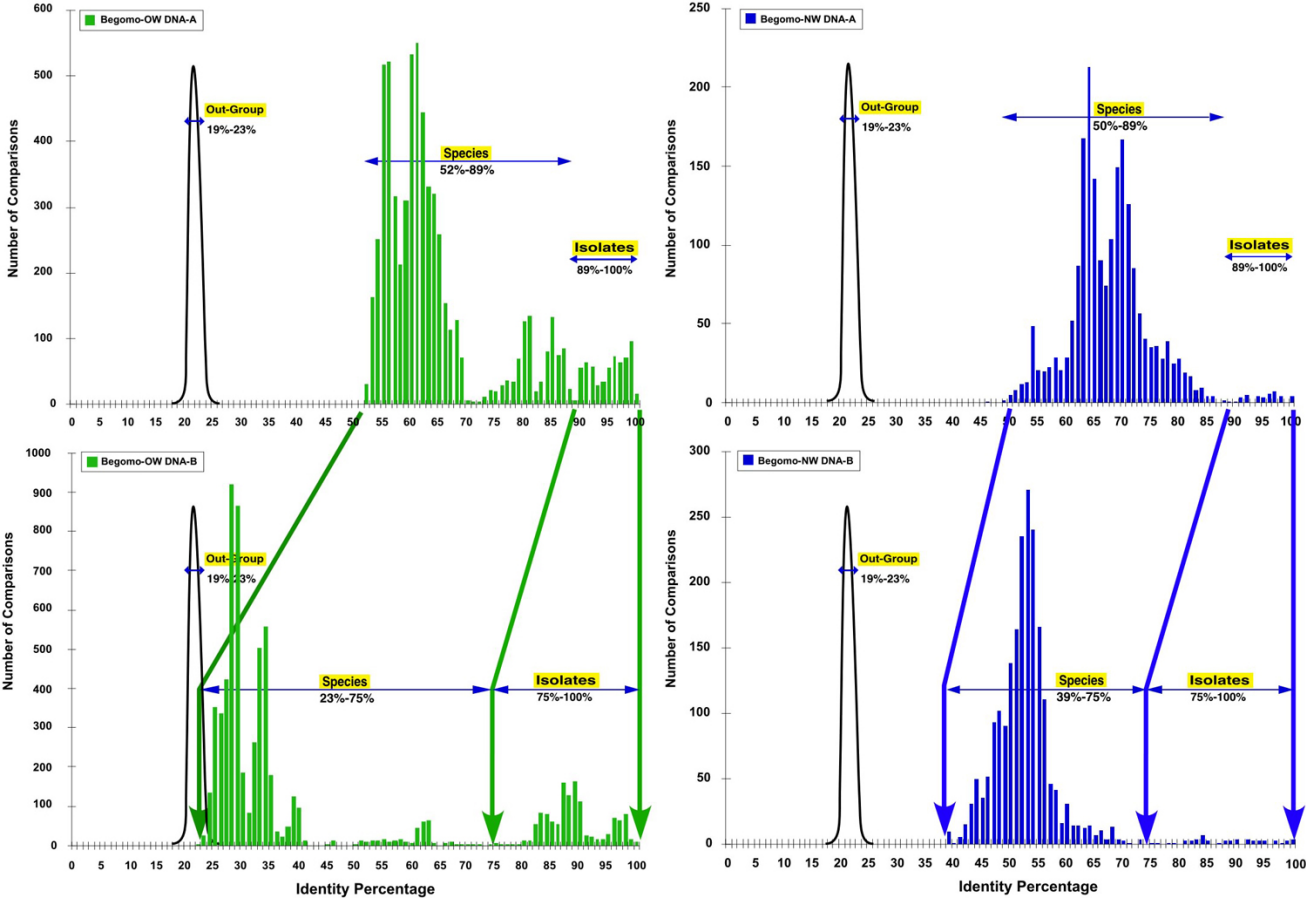
**Distribution of identity percentages of PASCs for the components of bipartite begomoviruses**. The percentage identity distribution of DNA-A components (top) or DNA-B components (bottom) of bipartite begomoviruses are shown. The diagrams on the left (green bars) represent comparisons for viruses from the OW, and the diagrams on the right (blue bars) are comparisons for viruses from the NW (with the corchoviruses). In addition, the black curves represent the position of the comparisons with CAV, an unrelated out-group.

A PASC analysis reveals that the DNA-A component sequences of 182 bipartite begomoviruses are distributed in the range 50 to 100% nucleotide sequence identity (Figure 4). The pairs between 50 and 89% represent comparisons between members of species, while the pairs between 89 and 100% represent comparisons between isolates of the same species [37]. The OW virus species cluster into two major peaks between 55 and 70% and between 70 and 89%, representing independent species and recombinant species respectively. It is noteworthy that the legumoviruses cluster in the lower values of the first peak (55-60%). The NW viruses also cluster into two peaks at 51 to 67% and 67 to 89%, with the OW-originating corchoviruses clustering in the lower range of the first peak (45-60%). These two clusters represent different groups of viruses in each part of the world.

### PASC analysis of DNA-B component sequences of bipartite begomoviruses

A PASC analysis reveals that the DNA-B component sequences of OW^- ^begomoviruses are scattered over a wide area of the diversity spectrum (Figure 4 lower panel). The members of species of the OW begomoviruses cluster between 24 and 41% while the recombinants cluster between 45 and 73%. The isolate comparisons cluster between 75 and 100%. The NW (with corchoviruses) DNA-B components form two peaks. A peak at 44 to 70% corresponds to comparisons between members of species whereas the second, at 73 to 100%, consists of comparisons between isolates. It is noteworthy that most of the species members from the OW do not cluster at the same identity percentage range as the members of the NW species. However, for both OW and NW viruses, the isolate comparisons fall between 75 and 100%. For simplicity, we considered the DNA-B "species" members as those associated with their cognate DNA-A species members according to the list of species recently defined [32]. Geminivirus species demarcation is based on both biological characteristics and sequence relatedness [1,32]. No separate classification system for DNA-B components is used, these being defined by their cognate DNA-A. This can lead to some confusion for the viruses that have exchanged DNA-B components.

### Comparison of the DNA-A and DNA-B component PASC distributions

A comparison of the range of sequence distributions for the DNA-A and DNA-B components, as well as a comparison of the change in distributions (between the components) for the NW (with the corchoviruses) and OW begomoviruses is shown in Table 1. The DNA-A component PASC distribution occupies a smaller range of sequence identities (40-100%) than the DNA-B distribution (24 to 100%). The NW and OW species occupy roughly the same range of sequence identities (54-89%). The values between 75 and 89% mostly represent recombinants that fill the gap between species and strains. The "isolate" peak for the DNA-A components divides into the "strains" at 87-93% and the "variants" between 94 and 100%.

**Table 1:** Comparison of DNA-A and DNA-B component PASC distributions.

	Species	OW species	NW species	Isolates	Strains	Variants
DNA-A components	40-89%	57-89%	54-85%	87-100%	87-93%	94-100%

DNA-B components	24-75%	24-73%	39-75%	75-100%	75-90%	91-100%

Change in identity percentage range between the DNA-A and DNA -B components	16-14%	**33-16%**	15-10%	12-0%	12-3%	3-0%

The DNA-B component pairwise distribution occupies a much larger range of sequence identities (24 to 100%), which is about 16% greater than that of the DNA-A components. The species comparisons occupy a space between 24 and 73%, which is comparable to the one occupied by species comparisons for the DNA-A component (49%), except that it is much lower in sequence identity (about 16% lower). The viruses of the species from the OW cluster in two distinct peaks, the first one from 24 to 42% and the second one from 53 to 73%. The species from the NW, in contrast, form a single peak which is skewed and covers a wider range of sequence identities (44 to 73%). In comparison to the isolates of component DNA-A, the isolates for component DNA-B also cover a greater range (up to 12%).

A comparison of the shift values between the DNA-A and DNA-B component pairwise distributions (Table 1) shows an increasing shift from the high percentages (90-100%) down to the low percentages (24-40%) with an increasing change from 3% to ~16%. However, for one of the two OW species, the shift is considerably larger (31 to 47%).

### Co-evolution of the DNA-A and DNA-B components

**Figure 5:**
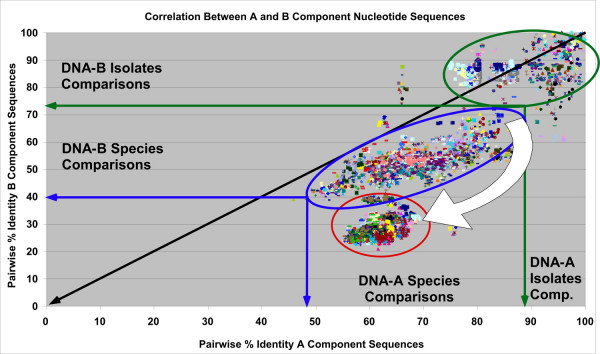
**Scatter plot analysis of the pairwise percentage identity values for the components of bipartite begomoviruses**. Scatter plots for cognate DNA-A (X axis) and DNA-B (Y axis) component sequences of bipartite begomoviruses are shown. For this analysis comparisons between NW and OW viruses were not included. The green oval (right) highlights isolate comparisons. The blue and red ovals highlight species representative's comparisons with most of the OW comparisons grouping at lower percentage identities (red oval). The white arrow is discussed in the text.

A total of 16,471 pairwise identity percentage values for comparisons of cognate DNA-A and DNA-B components of the 182 viruses considered were plotted on a scatter diagram (Figure 5). For the sake of clarity all comparisons (>10,000) of OW with NW viruses have been omitted. These all fall in the low percentage range and are not informative for this analysis.

**Figure 6:**
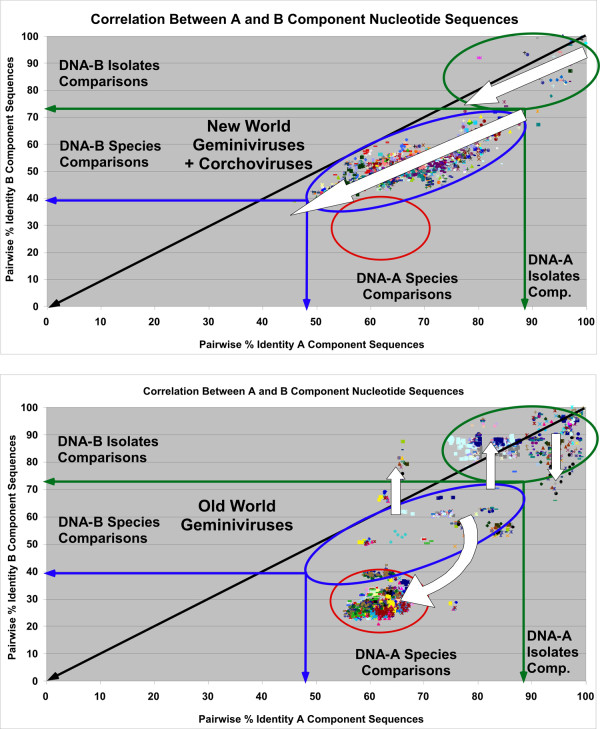
**Scatter plot analysis of the pairwise percentage identity values for begomoviruses from OW and NW**. Scatter plots for cognate DNA-A (X axis) and DNA-B (Y axis) component sequences of bipartite NW (with corchoviruses)(upper panel) and OW (lower panel) begomoviruses are shown. The green oval (right) highlights isolate comparisons. The blue and red ovals highlight species representative's comparisons with most of the OW comparisons grouping at lower percentage identities (red oval). The white arrows are discussed in the text.

If the DNA-A and DNA-B components had been under the same evolutionary pressure, all the points on the graph (Figure 5) would align along the diagonal. However the analysis shows a difference between the distributions of species and isolates. The isolates are scattered along the diagonal (green oval, Figure 5), whereas the species points fall well below the diagonal (blue oval) indicating a greater diversity of the DNA-B components. Furthermore the majority of OW viruses cluster tightly at very low DNA-B percentage values (left red oval), as is also evident in Figure 6. This indicates that, although OW DNA-B components are more diverse (covering a greater range of percentage identities) the majority of OW comparisons group within a smaller range of both DNA-A and DNA-B percentage identity values. This, and the apparent shift in DNA-B diversity away from the diagonal between isolate and species comparisons (indicated by the white arrow, Figure 5), indicates a differential evolution between the DNA-A and DNA-B components.

### Co-evolution of the DNA-A and DNA-B components of the NW viruses

**Figure 7:**
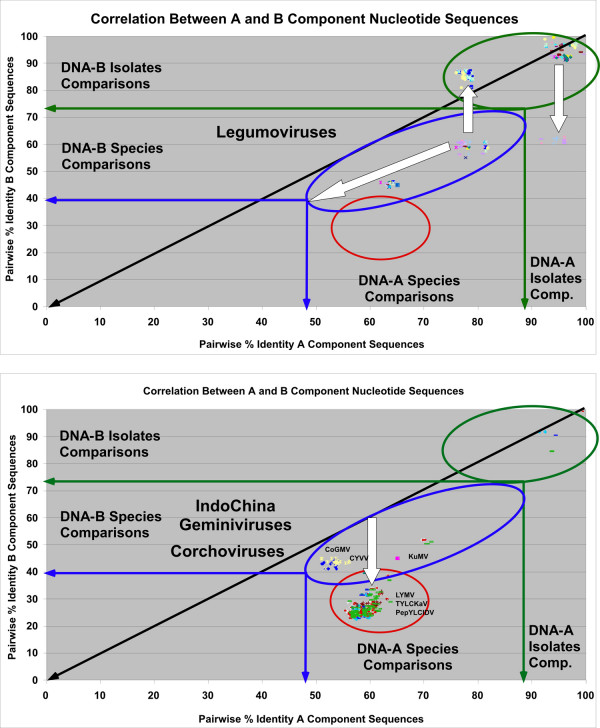
**Scatter plot analysis of the pairwise percentage identity values for legumoviruses and begomoviruses from Indo-China**. The pairwise percentage identity values for cognate DNA-A (X axis) and DNA-B (Y axis) component sequences of legumoviruses (upper panel) and bipartite begomoviruses originating from Indo-China (lower panel) are shown. The green oval (right) highlights isolate comparisons. The blue and red ovals highlight species representative's comparisons with most of the OW comparisons grouping at lower percentage identities (red oval). The white arrows are discussed in the text.

Figure 6 (upper panel) shows a co-evolution analysis of the DNA-A and DNA-B components of NW viruses. There is great homogeneity in the shift of all the DNA-B points relative to the DNA-A points, and the general trend is a change roughly parallel to the diagonal (indicated by the white arrows). Furthermore, it is interesting to note that there is a rough correlation between the phylogenetic tree organization and the percentage identity values for DNA-A and DNA-B component comparisons. The viruses belonging to the *Chino del tomate virus *cluster have the highest percentage identity values, while those belonging to the *Squash leaf curl virus *cluster have the lowest values of the NW viruses (data not shown) with only the corchoviruses having lower values (Figure 7, lower panel), mirroring their positions in the phylogenetic analysis relative to the NW viruses (Figure 1).

### Co-evolution of the DNA-A and DNA-B components of the OW viruses

Figure 6 (lower panel) shows a co-evolution analysis of the DNA-A and DNA-B components of OW viruses. In contrast to the NW virus comparisons, the OW viruses show a dramatic shift of the species points (relative to the isolate points) to very low percentages (indicated by the curved arrow), where most of the data points occur in a very small DNA-B percentage range (24-40%). There is also a change away from the diagonal for isolate and species comparisons and a vertical shift (indicated by the straight white arrows) for some of the DNA-B isolate data points, showing that the corresponding DNA-B components are simply exchanged between members of different species; thus with some DNA-A species having distinct DNA-B species as cognate DNA-B.

### Co-evolution of the DNA-A and DNA-B components of the OW legumoviruses

Figure 7 (upper panel) shows a co-evolution analysis for the components of legumoviruses. These viruses behave like typical OW viruses with the percentage identity shifts parallel to the diagonal (white arrow parallel to the diagonal). However, this group shows extensive evidence of pseudo-recombination (vertical white arrows).

### Co-evolution of the DNA-A and DNA-B component pairwise comparisons of the OW Indo-China viruses

Figure 7 (lower panel) shows the co-evolution of the DNA-A and DNA-B components of viruses isolated from Vietnam, Thailand and Indonesia that behave unusually in the phylogenetic analyses ("outsiders" in Figure 3). The Vietnamese viruses belonging to the NW cluster (CYVV and CoGMV) have a position typical of NW viruses, meaning a parallel shift to the diagonal, but very modest of about 10%. Kudzu mosaic virus (KuMV), the legumovirus isolated from Vietnam, also has a typical legumovirus position but with a larger shift of 20%, while the "outsiders", Pepper yellow leaf curl Indonesia virus (PepYLCIDV), Luffa yellow mosaic virus (LYMV) and Tomato yellow leaf curl Karnataka virus (TYLCKaV), behave like most of the OW viruses with a 35% shift.

### Taxonomy based on DNA-B component sequences

Until 1990, the point at which the first monopartite begomovirus sequences were published, the classification of begomoviruses was based on both genomic components. At this point it was self-evident that the classification of begomoviruses could only be based on the DNA-A (or homolog thereof for monopartite viruses) component. The analysis presented here allows us to examine, out of academic interest, what the classification of bipartite begomoviruses would be if it were based solely on the DNA-B component (Table 2). For 55 of the 65 species (based on DNA-A) represented, the classification would remain unchanged. Ten (DNA-A based) species would be downgraded to strains whereas one isolate would be upgraded to a new species. Thus the DNA-A-based classification system would remain ~85% unchanged, providing good support for the present classification based solely on the sequences of the DNA-A components.

**Table 2:** Taxon changes for a taxonomy based on DNA-B.

DNA-A Component	DNA-B component
65 species	55 species + 1 New species = **85%**

**Old World**	**28% changes**

EACMV	SACMV
	EACMKV
	EACMZV

SLCCNV	SLCPHV

SLCMV	ICMV

ToLCNDV	ToLCGV

**New World**	**12% changes**

CabLCuV	CabLCuJV

SiMoV	ToYSV

ToCMoMGV	ToCMoV-[MG].Bseq.AY090556

PYMV	PYMTV
	PYMPV

List of species that would be downgraded to strains and strains that would be upgraded to species for a classification based on DNA-B component sequences.

The analysis highlights the propensity of bipartite begomoviruses to exchange their DNA-B components (pseudo-recombination). One in six of the DNA-B components shows evidence of having been exchanged. For example, there are only seven begomovirus species in Africa and two in India that have been shown to cause cassava mosaic disease [42]. Based on DNA-B sequences there would be only four species. All the EACMV-like viruses (EACMV, EACMKV, EACMZV, EACMMV and SACMV), with the exception of EACMCV, would be considered a single species with four distinct strains. For the Asian viruses causing cassava mosaic disease, SLCMV and ICMV, there would be a single species with two strains. In addition there is not a simple correlation between what is a species based on DNA-A component sequence and what would be a strain based on DNA-B component sequence, due to component exchanges between species/strains. However, it is interesting to note that, apart from the example of Tomato chlorotic mottle virus (ToCMoV) from South America, all the strains within a species with the DNA-A-based classification remain in the same species with the DNA-B component-based classification, indicating that exchanges occurred prior to diversification to the strain level. Thus component exchange possibly drives diversification of species into strains, which is not surprising since DNA-B components play a major role in host range determination [43].

## Discussion

The geminiviruses as a whole have a very small range of genome (or genomic component) sizes; from 2550 nt for Cotton leaf crumple virus (*Begomovirus*) DNA-B to 3080 nt for the genome of Horseradish curly top virus (*Curtovirus*). This likely indicates that the capsid structure, consisting of 110 copies of a single protein (the CP) arranged into a quasi-icosahedral (geminate) structure as 22 capsomeres [44,45], has a finite capacity for ssDNA. Monomeric (icosahedral) capsids are reported for most purified virus preparations and likely encapsidate half unit length ssDNA molecules [46]. Multimeric particles (mainly trimers and tetramers) occur only infrequently, suggesting that they are not stable. In view of the congested nature of geminivirus genomes, with little if any scope for gaining additional functions (genes), the only option open to the monopartite progenitor of modern day bipartite begomoviruses was thus to expand genome size by adding an independently encapsidated DNA; either an additional genomic component or a satellite DNA.

The concept that geminiviruses have a modular arrangement is now well documented [47]. Thus certain elements of the genome are well adapted to work together, such as the MP and CP genes of mastreviruses [48] and the N-terminus of Rep (which interacts with the iterons that form part of the origin of replication [9,49-51]) and the origin of replication [47], and are thus less likely to be separated by recombination than distinct modules. It is clear here that DNA-B represents one such module, as evidenced by the relatively frequent exchange of this component for at least some of the groups of begomoviruses. Clearly maintaining this module as a separate unit in some circumstances facilitates its sexual exchange between distinct virus species and may represent an adaptation for diversification.

Why NW begomoviruses appear not to exchange DNA-B components with the high frequency demonstrated for their OW cousins is unclear. For this pseudo-recombination to occur, a single plant host cell would need to be infected by two viruses. It could also be conceivable that this could occur in the insect vector, assuming that the exchanged DNA-B can be transreplicated by its new DNA-A helper, since the two components of bipartite begomoviruses are encapsidated in separate geminate particles and there is no viral replication in vectors (precluding the recombination required for regulon grafting). The apparent absence of extensive component exchange in the NW could thus indicate that co-infections are (or at least were) not as frequent as in the OW. This, in turn, would suggest that the density of viruses in the NW is (or was) lower than in the OW. If, as has been suggested, begomoviruses were introduced into the NW only fairly recently, then a period of explosive speciation, following a limited introduction, could explain a low density of viruses. Alternatively, the low level of component exchange could be due to some mechanism (such as genetic isolation in a particular host due either to host specificity of the virus or the vector) that has prevented (or reduced) the frequency of co-infections. Experimentally NW begomoviruses appear to have few constraints to supporting pseudo-recombination, at least for related viruses [52,53]. However, there appear to be genetic differences between the (DNA-B-encoded) movement proteins of NW and OW begomoviruses. Frischmuth *et al*. [54] demonstrated that although the OW begomoviruses will mediate the systemic movement of the DNA-A components of NW viruses, the NW viruses cannot support the movement of the DNA-A components of OW begomoviruses - highlighting a further divergence between the NW and OW begomoviruses [55]. It is tempting to speculate that this difference is due to the absence of the V2 gene in NW viruses, as discussed later.

The similarities between the corchoviruses and begomoviruses occurring in the NW include the absence of the DNA-A-encoded V2 gene, the presence of a specific amino acid sequence motif in the CP, as well as the co-segregation of both components in phylogenetic analyses [33,34]. In the analyses conducted here the corchoviruses, originating from Vietnam, behave in all respects like the NW begomoviruses and this is consistent with the earlier reports. Ha *et al*. [33] suggested that the most likely explanation for this apparent presence of a NW-like virus in the OW was that all NW begomoviruses originated from a fairly recent introduction of begomoviruses into the NW, possibly by Chinese traders or much earlier by the Asian ancestors of the Amero-Indians. Certainly the relatively low level of diversity of the NW viruses is consistent with this hypothesis. However, the introduction would have needed to have been very limited, thus not include the monopartite and monopartite betasatellite-associated begomovirues. If this is the case, then it is possible that, at the time of the introduction, the NW-like begomoviruses were more widespread than at present (we note that recently CoGMV has been shown to occur in *Corchorus *in India [56]), or that the monopartite and monopartite betasatellite-associated begomoviruses evolved after the introduction - which seems unlikely - or were very limited in their geographic distribution at least until recently. It is clear that many questions about the origin of NW begomoviruses and their relationship to the OW viruses remain to be answered.

It has previously been argued that legumoviruses are (or at least were) genetically isolated in their legume hosts [15], as evidenced by the absence of recombination with other, non-leguminous begomoviruses. Possibly the corchoviruses have similarly been genetically isolated, preventing interaction with the more diverse begomoviruses infecting other plant species and shielding their unique NW-like characters from change. Why the corchoviruses and NW begomoviruses should show such a constrained and uniform pattern of genetic variation is a more vexing question. The possibility that the absence of the V2 gene is somehow responsible for this phenomenon should be investigated experimentally. The precise function of the V2 protein remains unclear although various investigations have shown it to have possible virus movement and suppression of gene silencing activity [30,57,58]. It is thus possible that the absence of these (or other as yet unknown) functions constrain variability. An investigation of the host-range of the corchoviruses might provide some answers since, if these viruses are genetically isolated (presumably in their natural host jute [*Corchorus capsularis*]), the absence of V2 may be an adaptation to this host.

From the comparisons of the pairwise distributions of the DNA-A and DNA-B component sequences of begomoviruses, it is evident that they have followed different evolutionary paths and have experienced different evolutionary pressures, or have responded differently to the evolutionary pressures. The DNA-B components show a much wider range of molecular diversity than the DNA-A components [59]. The reasons for this are unclear. Possibly the DNA-B component, by virtue of it not encoding overlapping genes, has a greater capacity for variation. The ranges of interactions this component's gene products undergo may be fewer or may be less constraining. The highly specialized nature of the DNA-B component gene products, which interact with plasmodesmata and nuclear pores to mediate cell-to-cell and long distance movement, may allow for a greater range of variation than is possible with the DNA-A component which is more modular and has more cis- (including transactivation, Rep-iteron recognition, Rep-REn interaction) and trans-interactions (including transactivation, Rep-iteron recognition, CP-vector interaction) to maintain. Alternatively, the differences may indicate that the genomes of the majority of OW bipartite begomoviruses result from component exchange. It is noticeable that there are relatively few bipartite begomoviruses in the OW, or at least only few have been identified, and the majority of the ones with unusual behaviour in the PASC analyses are isolated from introduced crops (including cassava, tomato and peppers) whereas those that might be considered as being isolated from native species (such as *Corchorus *and the legume-infecting viruses), and thus might be considered more ancient, have a behaviour more in line with those from the NW and more in-line with what would be expected were the two components under similar evolutionary pressures. What the PASC analysis may be showing could thus be the result of relatively young DNA-A-DNA-B interactions, for which the DNA B component has yet to be fully shaped by the relationship.

The analyses of bipartite begomovirus component sequences indicate that our present classification system, based entirely on their DNA-A components, holds for the majority of viruses were the system instead to be based on DNA-B components; the exceptions to this being the cases where pseudo-recombination has occurred. There would thus be little to gain from including DNA-B sequences at the species level. Possibly this character should be relegated to the strain level; thus two isolates of a species with distinct DNA-Bs might be designated distinct strains. In view of the fact that DNA-B components play a significant role in host range determination, this may be a desirable option. However, although this might be useful in some cases, it would require a separate classification system for the DNA-B components which might prove too cumbersome.

This overall very good similarity shows that most of the DNA-As and DNA-Bs of bipartite geminiviruses of the same species have been associated for a very long time and had the same type of evolutionary pressure. Nevertheless, a significant number of viruses show evidence of component exchange by a mechanism known as regulon grafting. The fact that geminiviruses have the possibility to recombine easily [17,18,60-65] increases the chances of a DNA-A component CR introgression in a non-related DNA-B component, to make that DNA-B component dependent on the replication of the cognate DNA-A component. There is evidence of the natural occurrence of the recombination between the helper begomovirus genomes and their betasatellite molecules that supports this hypothesis [18,66]. There is also the sequence analysis evidence for PYMV isolates (from Panama, Venezuela and Trinidad) where the same CR has been introgressed simultaneously in DNA-A and DNA-B components having over all very different sequences (C. Fauquet, unpublished results). In this latter case a dual recombination had to occur at the same time to recreate a viable strict bipartite geminivirus, and this happened at least two times, once with the PYMV isolate from Trinidad and Tobago, and once with the PYMPV isolate from Panama. It is conceivable that the synergism between two molecules such as a geminivirus and a betasatellite molecule based on mutual biological gain, can be maintained over very long periods of time [67], but it is also more intuitively acceptable that the physical interdependence of replication between two molecules be a more stringent level of interdependence in virus evolution. It is interesting to note that geminivirus satellites have so far been found almost exclusively with monopartite begomoviruses. The exceptions are TYLCTHV, which is an unusual virus with a DNA-A which is infectious to plants (experimentally in the absence of DNA-B) but occurs in the field with either a DNA-B or a DNA-β [19,68], ToLCNDV and MYMIV [69]; the nature of the interaction of the latter two viruses with betasatellites has not been investigated. Possibly this indicates that the presence of a DNA-B component prevents/interferes with the interaction of the DNA-A component with a satellite.

Comparisons of the PASC distributions of DNA-A and DNA-B components show a fundamentally different behaviour between the NW and OW viruses. NW viruses show a very homogenous shift of their DNA-B component sequences towards a lower percentage of identity, paralleling the diagonal that represents a quasi-perfect co-evolution. In contrast, the OW viruses fall into two groups with distinct behaviours. For most OW viruses there is a dramatic shift (30 to 47%) in the PASC percentage identity values between DNA-A and DNA-B components, bringing the values close to comparison with a random sequence. For a small number of OW viruses the change is more modest (± 10%), similar to that seen with the NW viruses. This may indicate that some of the OW viruses have been under entirely different selection pressures. The most obvious difference between OW and NW begomoviruses is the absence of the AV2 gene in the latter. The AV2 protein has been shown to play a role in the movement of begomoviruses [57] and possibly also, either directly or indirectly, in the expression of the CP [70], which is itself involved in movement. It is thus possible that OW begomoviruses, having an AV2, have more flexibility with regard to movement *in planta*. As well as allowing them to, in some cases, spread and cause disease in the absence of a DNA-B, it may allow them to additionally interact with other molecules, such as betasatellites and, more readily than for the NW viruses, the DNA-B components of other species. However, it is difficult to see, knowing what we do about the functions encoded by AV2, how this gene might predispose a virus to component exchange.

Satellites are a common feature of a number of RNA viruses and also begomoviruses [5,71]. These molecules are classically defined as viruses or nucleic acids that depend on a helper virus for replication, are dispensable for the replication of the helper virus, and lack any appreciable sequence similarity to the helper virus' genome [72]. With the exception of the CR, begomovirus DNA-B components are essentially sequence unrelated to their cognate DNA-A components. Thus DNA-B components satisfy all except one of the criteria of satellites. The presently accepted hypothesis for the origin of DNA-B contends that it originated by component duplication of the DNA-A, followed by gain of a new gene (the movement protein encoded in the complementary-sense) from an unknown source and possible divergence of the CP to become the nuclear shuttle protein (NSP; encoded in the virion-sense). Although Kikuno *et al*. [73] reported some similarity between the amino acid sequences of the CP and NSP sequences for ACMV, this has not been borne-out by modern sequence analysis software applied to either ACMV or the much larger sample set of bipartite begomovirus sequences now available (C.M. Fauquet, unpublished results). However, an equally plausible hypothesis is that the begomovirus DNA-B component originated as a satellite captured by the monopartite progenitor of all bipartite begomoviruses in the same way that betasatellites and alphasatellites were. Although the origins of the betasatellites remain uncertain, the alphasatellites almost certainly originated with the nanoviruses, which themselves are helper viruses of a related class of alphasatellites [6]. The satellite progenitor of modern DNA-B components would have needed to provide its helper begomoviruses with some selective advantage for it to be maintained. In most cases the betasatellites allow their helper begomoviruses to productively infect hosts to which they are otherwise only poorly infectious [74,75], either by overcoming host defenses [76] or possibly by providing additional movement functions [77]. It is thus possible that the selective advantage provided by the early DNA-B was more efficient movement in plants, possibly in the form of the MP. The satellite would then have been domesticated into a *bona fide *genome component by regulon grafting from DNA-A (for which there is extensive evidence in both the DNA-Bs and betasatellites, as detailed earlier) and possible gene duplication (of the CP to yield the NSP) leading to the DNA-B component we recognize today.

For the bipartite begomoviruses the genes encoded by DNA-B have been shown to play a major part in controlling genome size. Unit length (~2800 nt) viral DNA is moved cell-to-cell more effectively than larger molecules and larger DNA molecules undergo deletion to restore approximately unit size length [78]. This size selection is mediated by MP which has evolved to usurp the non-cell autonomous RNA trafficking pathway through plasmodesmata. The mechanism for maintaining genome size of monopartite viruses is less clear. Frischmuth *et al*. [46] have shown, for the bipartite begomovirus ACMV, that genome size is a determining factor in particle multiplicity; thus half unit length molecules (such as diDNAs and probably betasatellites and alphasatellites) are encapsidated in monomeric (icosahedral) particles whereas unit length molecules are encapsidated in geminate particles and larger molecules in multimeric particles. The relative rarity with which multimeric particles are encountered possibly suggests that these are unstable. Thus packaging constraints may be an important control of geminivirus genome size, particularly for the monopartite viruses. For the begomovirus-associated beta- and alphasatellites there is some circumstantial evidence of a need to satisfy a size constraint. The presence in both of an adenine (A)-rich stretch of sequence has been taken to signify an increase in size over the progenitor component (the only real evidence for this being the lack of a similar A-rich sequence in the smaller nanovirus components, the closest relatives of the alphasatellites [65,79], although this does not explain why the sequence needs to be rich in adenine). The evolutionary maintenance by DNA-B components of a size close to that of their DNA-A "helper", when it is clear that a half unit length size can be accommodated, may indicate either that there are coding constraints to having the MP and NSP in overlapping reading frames (which would be required were they to be encoded by a half unit length molecule), or more likely that there is a need for strict spatial and temporal control of the genes during infection, which is possible when they are under the control of distinct promoters (the NSP gene being inducible by TrAP [80]). Little is known about the control of expression of the βC1 gene encoded by betasatellites. Available evidence suggests it is controlled by a strong promoter which is constitutive and phloem specific [81,82].

## Conclusion

The analysis shows that the DNA-B components of bipartite begomoviruses are far more diverse than their DNA-A partners. This may be due to the DNA-B component being less congested (encoding fewer functions and thus being more permissive of variation), the DNA-B component evolving exclusively in response to the host (whereas the DNA-A has to additionally maintain interaction with the arthropod vector) or, as we propose for the first time, that DNA-B has an origin that is distinct from DNA-A (possibly originating as a captured and domesticated satellite). The most likely explanation for the difference is that a combination of these factors is responsible. Pseudorecombination is shown to have occurred much more frequently than previously recognized and to be a property almost exclusively exhibited by OW begomoviruses. Although the evidence is only circumstantial, the presence in OW begomoviruses of an additional movement function (the V2 gene) may be the factor facilitating this phenomenon. The co-evolution study for the components of the genomes of bipartite begomoviruses highlights distinct differences between the majority of OW viruses and a group that includes all the NW begomoviruses and a small number of OW viruses. The latter group shows near perfect co-evolution of their DNA-A and DNA-B components whereas for the majority of the OW viruses this is not the case. This indicates that for the majority of OW bipartite begomoviruses the DNA-A and DNA-B components have been under distinct evolutionary pressures or have responded differently to the pressure. Since the majority of OW begomoviruses with uniform DNA-A/DNA-B behaviour occur in native plant species, whereas the ones with unusual behaviour (thus most of the OW viruses) occur in introduced species, it is possible that this dichotomy is due to the host and due to pesudorecombination, suggesting a recent and immature DNA-A/DNA-B interaction.

## Methods

### Sequences analysed

The sequences of the genomes (or DNA-A components) of 389 geminiviruses were downloaded from GenBank. The dataset contains the full-length sequences of the cognate DNA-A and DNA-B components of 182 bipartite begomoviruses. For several sequences corrections have been made as indicated previously [31]. For brevity only the standardised virus acronyms will be used [1,32].

### Pairwise sequence comparison and phylogenetic analysis

All possible pairwise sequence comparison (PASC) percentage identities were plotted as frequency distributions to examine the distributions within and between taxa/subgroups [83].

Sequences were aligned using the Clustal V method of aligning multiple sequences using MegAlign (v. 3.11) available in the Lasergene sequence analysis package (v.1.02 for the Apple Computer; DNASTAR Inc.). A random sequence of equal length and composition was included in all alignments to show pairwise percent identities that are not significantly different from random identity. For phylogenetic studies, the sequence of Chicken anemia virus (CAV; accession number M55918), a member of the genus *Circovirus *in the family *Circoviridae*, was used as an out-group.

Phylogenetic analyses were conducted using the cladistic parsimony method and the program PAUP (version 3.1.1 [84]). Optimum trees were obtained with the heuristic method using the tree-bisection-reconnection branch-swapping option. One hundred bootstrap replications were performed to place confidence estimates on groups contained in the most parsimonious tree. Phylogenetic analyses were also conducted using the UPGMA distance matrix and neighborhood joining method available with the MegAlign program. In this case a preliminary phylogeny is derived from the distance between pairs of input sequences and the application of the UPGMA algorithm that guides the alignment of ancestral sequences [85]. The final phylogeny was obtained by applying the neighborhood joining method to the distance and alignment data [86]. The trees generated by both PAUP and MegAlign were nearly identical, and the tree presented here was generated using the MegAlign program.

## Competing interests

The authors declare that they have no competing interests.

## Authors' contributions

RB wrote the paper and contributed to the Discussion. BP contributed to the Introduction, Discussion and Results. BB contributed to the elaboration of the paper. MSN downloaded sequences, made their phylogenies, calculated similarity percentages of different DNA-B components and contributed to the elaboration of the paper. CMF conceived of the study, performed the sequence and phylogenetic analyses, created the figures, wrote the paper and contributed to the Discussion. All authors have read and approved the final manuscript.

## Supplementary Material

Additional file 1 **Figure S1**. Phylogenetic trees constructed from an alignment of DNA-A (A) and DNAB (B) component sequences of 182 bipartite begomoviruses. The trees were constructed from 2947 informative sites for the DNA-A alignment and 3223 informative sites for the DNA-B alignment. For brevity only the standardised virus acronyms are used [1,32] complemented by the database accession numbers. Sequences were aligned using the Clustal V method of aligning multiple sequences using MegAlign (v. 3.11) available in the Lasergene sequence analysis package (v.1.02 for the Apple Computer; DNASTAR Inc.). Phylogenetic analyses were conducted using the UPGMA distance matrix and neighborhood joining method available with the MegAlign program. The dashed branches indicate a bootstrap value below 50% and the scale below the tree indicates the distance between sequences. Click here for file
