# Supplementary material for: Distinct evolutionary histories of the DNA-A and DNA-B components of bipartite begomoviruses
Source: BMC Evol Biol. 2010 Apr 8;10:97. doi: 10.1186/1471-2148-10-97 (PMC2858149; doi:10.1186/1471-2148-10-97)
Supplement: Additional file 1 — Figure S1. Phylogenetic trees constructed from an alignment of DNA-A (A) and DNAB (B) component sequences of 182 bipartite begomoviruses. The trees were constructed from 2947 informative sites for the DNA-A alignment and 3223 informative sites for the DNA-B alignment. For brevity only the standardised virus acronyms are used [1,32] complemented by the database accession numbers. Sequences were aligned using the Clustal V method of aligning multiple sequences using MegAlign (v. 3.11) available in the Lasergene sequence analysis package (v.1.02 for the Apple Computer; DNASTAR Inc.). Phylogenetic analyses were conducted using the UPGMA distance matrix and neighborhood joining method available with the MegAlign program. The dashed branches indicate a bootstrap value below 50% and the scale below the tree indicates the distance between sequences. [file 1471-2148-10-97-S1.PDF]

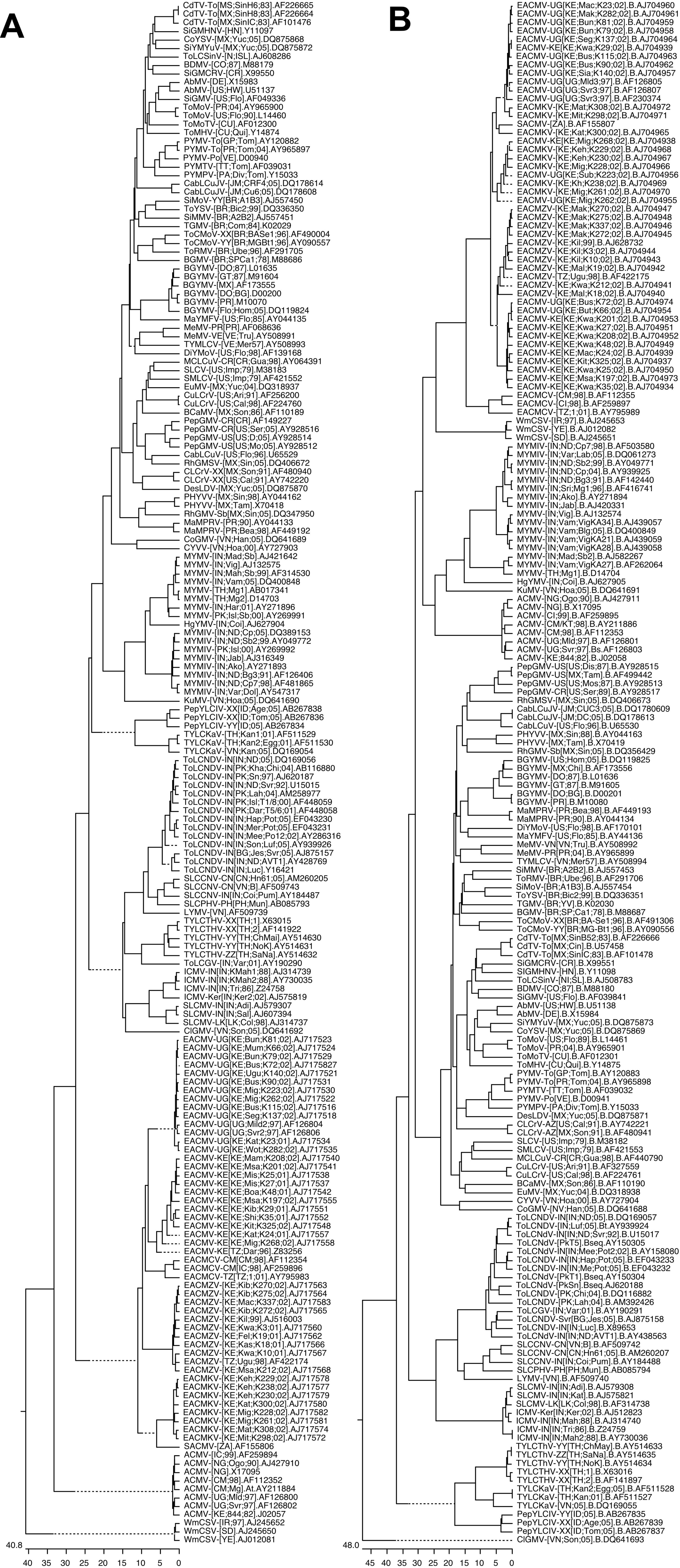

A
CdTV-To[MS;SinH6;83].AF226665
CdTV-To[MX;SinH8;83].AF226664
CdTV-To[MX;SinIC;83].AF101476
SiGMHNV-[HN].Y11097
CoYSV-[MX;Yuc;05].DQ875868
SiYMYuV-[MX;Yuc;05].DQ875872
ToLCSinV-[N;ISL].AJ608286
BDMV-[CO;87].M88179
SiGMCRV-[CR].X99550
AbMV-[DE].X15983
AbMV-[US;HW].U51137
SiGMV-[US;Flo].AF049336
ToMoV-[PR;04].AY965900
ToMoV-[US;Flo;90].L14460
ToMoTV-[CU].AF012300
ToMHV-[CU;Qui].Y14874
PYMV-To[GP;Tom].AY120882
PYMV-To[PR;Tom;04].AY965897
PYMV-Po[VE].D00940
PYMTV-[TT;Tom].AF039031
PYMPV-[PA;Div;Tom].Y15033
CabLCuJV-[JM;CRF4;05].DQ178614
CabLCuJV-[JM;Cu6;05].DQ178608
SiMoV-YY[BR;A1B3].AJ557450
ToYSV-[BR;Bic2;99].DQ336350
SiMMV-[BR;A2B2].AJ557451
TGMV-[BR;Com;84].K02029
ToCMoV-XX[BR;BASe1;96].AF490004
ToCMoV-YY[BR;MGBt1;96].AY090557
ToRMV-[BR;Ube;96].AF291705
BGMV-[BR;SPCa1;78].M88686
BGYMV-[DO;87].L01635
BGYMV-[GT;87].M91604
BGYMV-[MX].AF173555
BGYMV-[DO;BG].D00200
BGYMV-[PR].M10070
BGYMV-[Flo;Hom;05].DQ119824
MaYMFV-[US;Flo;85].AY044135
MeMV-PR[PR].AF068636
MeMV-VE[VE;Tru].AY508991
TYMLCV-[VE;Mer57].AY508993
DiYMoV-[US;Flo;98].AF139168
MCLCuV-CR[CR;Gua;98].AY064391
SLCV-[US;Imp;79].M38183
SMLCV-[US;Imp;79].AF421552
EuMV-[MX;Yuc;04].DQ318937
CuLCrV-[US;Ari;91].AF256200
CuLCrV-[US;Cal;98].AF224760
BCaMV-[MX;Son;86].AF110189
PepGMV-CR[CR].AF149227
PepGMV-CR[US;Ser;05].AY928516
PepGMV-US[US;D;05].AY928514
PepGMV-US[US;Mo;05].AY928512
CabLCuV-[US;Flo;96].U65529
RhGMSV-[MX;Sin;05].DQ406672
CLCrV-XX[MX;Son;91].AF480940
CLCrV-XX[US;Cal;91].AY742220
DesLDV-[MX;Yuc;05].DQ875870
PHYVV-[MX;Sin;98].AY044162
PHYVV-[MX;Tam].X70418
RhGMV-Sb[MX;Sin;05].DQ347950
MaMPRV-[PR;90].AY044133
MaMPRV-[PR;Bea;98].AF449192
CoGMV-[VN;Han;05].DQ641689
CYVV-[VN;Hoa;00].AY727903
MYMV-[IN;Mad;Sb].AJ421642
MYMV-[IN;Vig].AJ132575
MYMV-[IN;Mah;Sb;99].AF314530
MYMV-[IN;Vam;05].DQ400848
MYMV-[TH;Mg1].AB017341
MYMV-[TH;Mg2].D14703
MYMV-[IN;Har;01].AY271896
MYMV-[PK;Isl;Sb;00].AY269991
HgYMV-[IN;Coi].AJ627904
MYMIV-[IN;ND;Cp;05].DQ389153
MYMIV-[IN;ND;Sb2;99.AY049772
MYMIV-[PK;Isl;00].AY269992
MYMIV-[IN;Jab].AJ316349
MYMIV-[IN;Ako].AY271893
MYMIV-[IN;ND;Bg3;91].AF126406
MYMIV-[IN;ND;Cp7;98].AF481865
MYMIV-[IN;Var;Dol].AY547317
KuMV-[VN;Hoa;05].DQ641690
PepYLCIV-XX[ID;Age;05].AB267838
PepYLCIV-XX[ID;Tom;05].AB267836
PepYLCIV-YY[ID;05].AB267834
TYLCKaV-[TH;Kan1;01].AF511529
TYLCKaV-[TH;Kan2;Egg;01].AF511530
TYLCKaV-[VN;Kan;05].DQ169054
ToLCNDV-IN[IN;ND;05].DQ169056
ToLCNDV-IN[PK;Kha;Chi;04].AB116880
ToLCNDV-IN[PK;Sn;97].AJ620187
ToLCNDV-IN[IN;ND;Svr;92].U15015
ToLCNDV-IN[PK;Lah;04].AM258977
ToLCNDV-IN[PK;Isl;T1/8;00].AF448059
ToLCNDV-IN[PK;Dar;T5/6;01].AF448058
ToLCNDV-IN[IN;Hap;Pot;05].EF043230
ToLCNDV-IN[IN;Mer;Pot;05].EF043231
ToLCNDV-IN[IN;Mee;Po12;02].AY286316
ToLCNDV-IN[IN;Son;Luf;05].AY939926
ToLCNDV-IN[BG;Jes;Svr;05].AJ875157
ToLCNDV-IN[IN;ND;AVT1].AY428769
ToLCNDV-IN[IN;Luc].Y16421
SLCCNV-CN[CN;Hn61;05].AM260205
SLCCNV-CN[VN;B].AF509743
SLCCNV-IN[IN;Coi;Pum].AY184487
SLCPHV-PH[PH;Mun].AB085793
LYMV-[VN].AF509739
TYLCTHV-XX[TH;1].X63015
TYLCTHV-XX[TH;2].AF141922
TYLCTHV-YY[TH;ChMai].AY514630
TYLCTHV-YY[TH;NoK].AY514631
TYLCTHV-ZZ[TH;SaNa].AY514632
ToLCGV-[IN;Var;01].AY190290
ICMV-IN[IN;KMah1;88].AJ314739
ICMV-IN[IN;KMah2;88].AY730035
ICMV-IN[IN;Tri;86].Z24758
ICMV-Ker[IN;Ker2;02].AJ575819
SLCMV-IN[IN;Adi].AJ579307
SLCMV-IN[IN;Sal].AJ607394
SLCMV-LK[LK;Col;98].AJ314737
ClGMV-[VN;Son;05].DQ641692
EACMV-UG[KE;Bun;K81;02].AJ717523
EACMV-UG[KE;Mum;K66;02].AJ717524
EACMV-UG[KE;Bun;K79;02].AJ717529
EACMV-UG[KE;Bus;K72;02].AJ7175827
EACMV-UG[KE;Ugu;K140;02].AJ717521
EACMV-UG[KE;Bus;K90;02].AJ717531
EACMV-UG[KE;Mig;K223;02].AJ717530
EACMV-UG[KE;Mig;K262;02].AJ717522
EACMV-UG[KE;Bus;K115;02].AJ717578
EACMV-UG[KE;Seg;K137;02].AJ717518
EACMV-UG[UG;Mld2;97].AF126804
EACMV-UG[UG;Svr2;97].AF126806
EACMV-UG[KE;Kat;K23;01].AJ717534
EACMV-UG[KE;Wot;K282;02].AJ717535
EACMV-KE[KE;Mam;K208;02].AJ717540
EACMV-KE[KE;Msa;K201;02].AJ717541
EACMV-KE[KE;Mis;K25;01].AJ717538
EACMV-KE[KE;Mis;K27;01].AJ717537
EACMV-KE[KE;Boa;K48;01].AJ717542
EACMV-KE[KE;Msa;K197;02].AJ717555
EACMV-KE[KE;Kib;K29;01].AJ717551
EACMV-KE[KE;Shi;K35;01].AJ717552
EACMV-KE[KE;Kit;K325;02].AJ717548
EACMV-KE[KE;Kat;K24;01].AJ717557
EACMV-KE[KE;Mig;K268;02].AJ717558
EACMV-KE[TZ;Dar;96].Z83256
EACMCV-CM[CM;98].AF112354
EACMCV-CM[IC;98].AF259896
EACMCV-TZ[TZ;1;01].AY795983
EACMZV-[KE;Kib;K270;02].AJ717563
EACMZV-[KE;Kib;K275;02].AJ717564
EACMZV-[KE;Mac;K337;02].AJ717583
EACMZV-[KE;Kib;K272;02].AJ717565
EACMZV-[KE;Kil;99].AJ516003
EACMZV-[KE;Kwa;K3;01].AJ717560
EACMZV-[KE;Fel;K19;01].AJ717562
EACMZV-[KE;Kas;K18;01].AJ717566
EACMZV-[KE;Kwa;K10;01].AJ717567
EACMZV-[TZ;Ugu;98].AF422174
EACMZV-[KE;Msa;K212;02].AJ717568
EACMKV-[KE;Keh;K229;02].AJ717578
EACMKV-[KE;Keh;K238;02].AJ717577
EACMKV-[KE;Keh;K230;02].AJ717579
EACMKV-[KE;Kat;K300;02].AJ717580
EACMKV-[KE;Mig;K228;02].AJ717582
EACMKV-[KE;Mig;K261;02].AJ717581
EACMKV-[KE;Mat;K308;02].AJ717574
EACMKV-[KE;Mit;K298;02].AJ717572
SACMV-[ZA].AF155806
ACMV-[IC;99].AF259894
ACMV-[NG;Ogo;90].AJ427910
ACMV-[NG].X17095
ACMV-[CM;98].AF112352
ACMV-[CM;Mg].At.AY211884
ACMV-[UG;Mld;97].AF126800
ACMV-[UG;Svr;97].AF126802
ACMV-[KE;844;82].J02057
WmCSV-[IR;97].AJ245652
WmCSV-[SD].AJ245650
WmCSV-[YE].AJ012081
40.8
40
35
30
25
20
15
10
5
0
B
EACMV-UG[KE;Mac;K23;02].B.AJ704960
EACMV-UG[KE;Mak;K282;02].B.AJ704961
EACMV-UG[KE;Bun;K81;02].B.AJ704959
EACMV-UG[KE;Bun;K79;02].B.AJ704958
EACMV-UG[KE;Seg;K137;02].B.AJ704964
EACMV-KE[KE;Kwa;K29;02].B.AJ704939
EACMV-UG[KE;Bus;K115;02].B.AJ704963
EACMV-UG[KE;Bus;K90;02].B.AJ704962
EACMV-UG[KE;Sia;K140;02].B.AJ704957
EACMV-UG[UG;Mld3;97].B.AF126805
EACMV-UG[UG;Svr3;97].B.AF126807
EACMV-UG[UG;Svr3;97].B.AF230374
EACMKV-[KE;Mat;K308;02].B.AJ704972
EACMKV-[KE;Mit;K298;02].B.AJ704971
SACMV-[ZA].B.AF155807
EACMKV-[KE;Kat;K300;02].B.AJ704965
EACMKV-KE[KE;Mig;K268;02].B.AJ704938
EACMKV-[KE;Keh;K229;02].B.AJ704968
EACMKV-[KE;Keh;K230;02].B.AJ704967
EACMKV-[KE;Mig;K228;02].B.AJ704966
EACMKV-UG[KE;Sub;K223;02].B.AJ704956
EACMKV-[KE;Kh;K238;02].B.AJ704969
EACMKV-[KE;Mig;K261;02].B.AJ704970
EACMV-UG[KE;Mig;K262;02].B.AJ704955
EACMZV-[KE;Mak;K270;02].B.AJ704947
EACMZV-[KE;Mak;K275;02].B.AJ704948
EACMZV-[KE;Mak;K337;02].B.AJ704946
EACMZV-[KE;Mak;K272;02].B.AJ704945
EACMZV-[KE;Kil;99].B.AJ628732
EACMZV-[KE;Kil;K3;02].B.AJ704944
EACMZV-[KE;Kil;K10;02].B.AJ704943
EACMZV-[KE;Mal;K19;02].B.AJ704942
EACMZV-[TZ;Ugu;98].B.AF422175
EACMZV-[KE;Kwa;K212;02].B.AJ704941
EACMZV-[KE;Mal;K18;02].B.AJ704940
EACMV-UG[KE;Bus;K72;02].B.AJ704974
EACMV-UG[KE;But;K66;02].B.AJ704954
EACMV-KE[KE;Kwa;K201;02].B.AJ704953
EACMV-KE[KE;Kwa;K27;02].B.AJ704951
EACMV-KE[KE;Kwa;K208;02].B.AJ704952
EACMV-KE[KE;Kwa;K48;02].B.AJ704949
EACMV-KE[KE;Mac;K24;02].B.AJ704939
EACMV-KE[KE;Kit;K325;02].B.AJ704937
EACMV-KE[KE;Kwa;K25;02].B.AJ704950
EACMV-KE[KE;Msa;K197;02].B.AJ704973
EACMV-KE[KE;Kwa;K35;02].B.AJ704934
EACMCV-[CM;98].B.AF112355
EACMCV-[CI;98].B.AF259897
EACMCV-[TZ;1;01].B.AY795989
WmCSV-[IR;97].B.AJ245653
WmCSV-[YE].B.AJ012082
WmCSV-[SD].B.AJ245651
MYMIV-[IN;ND;Cp7;98].B.AF503580
MYMIV-[IN;Var;Lab;05].B.DQ061273
MYMIV-[IN;ND;Sb2;99].B.AY049771
MYMIV-[IN;ND;Cp;04].B.AY939925
MYMIV-[IN;ND;Bg3;91].B.AF142440
MYMIV-[IN;Sri;Mg1;96].B.AF416741
MYMIV-[IN;Ako].B.AY271894
MYMIV-[IN;Jab].B.AJ420331
MYMV-[IN;Vig].B.AJ132574
MYMV-[IN;Vam;VigKA34].B.AJ439057
MYMV-[IN;Blg;05].B.DQ400849
MYMV-[IN;Vam;VigKA21].B.AJ439059
MYMV-[IN;Vam;VigKA28].B.AJ439058
MYMV-[IN;Mad;Sb2].B.AJ582267
MYMV-[IN;Vam;VigKA27].B.AF262064
MYMV-[TH;Mg1].B.D14704
HgYMV-[IN;Coi].B.AJ627905
KuMV-[VN;Hoa;05].B.DQ641691
ACMV-[NG;Ogo;90].B.AJ427911
ACMV-[NG].B.X17095
ACMV-[CI;99].B.AF259895
ACMV-[CM/KT;98].B.AY211886
ACMV-[CM;98].B.AF112353
ACMV-[UG;Mld;97].B.AF126801
ACMV-[UG;Svr;97].Bs.AF126803
ACMV-[KE;844;82].B.J02058
PepGMV-US[US;Dis;87].B.AY928515
PepGMV-US[MX;Tam].B.AF499442
PepGMV-US[US;Mos;87].B.AY928513
PepGMV-CR[US;Ser;89].B.AY928517
RhGMSV-[MX;Sin;05].B.DQ406673
CabLCuJV-[JM;CUC3;05].B.DQ1780609
CabLCuJV-[JM;DC;05].B.DQ178613
CabLCuV-[US;Flo;96].B.U65530
PHYVV-[MX;Sin;88].B.AY044163
PHYVV-[MX;Tam].B.X70419
RhGMV-Sb[MX;Sin;05].B.DQ356429
BGYMV-[US;Hom;05].B.DQ119825
BGYMV-[MX;Chi].B.AF173556
BGYMV-[DO;87].B.L01636
BGYMV-[GT;87].B.M91605
BGYMV-[DO;BG].B.D00201
BGYMV-[PR].B.M10080
MaMPRV-[PR;Bea;98].B.AF449193
MaMPRV-[PR;90].B.AY044134
DiYMoV-[US;Flo;98].B.AF170101
MaYMFV-[US;Flo;85].B.AY44136
MeMV-VN[VN;Tru].B.AY508992
MeMV-PR[PR].B.AY965899
TYMLCV-[VN;Mer57].B.AY508994
SiMMV-[BR;A2B2].B.AJ557453
ToRMV-[BR;Ube;96].B.AF291706
SiMoV-[BR;A1B3].B.AJ557454
ToYSV-[BR;Bic2;99].B.DQ336351
TGMV-[BR;YV].B.K02030
BGMV-[BR;SP;Ca1;78].B.M88687
ToCMoV-XX[BR;BA-Se1;96].B.AF491306
ToCMoV-YY[BR;MG-Bt1;96].B.AY090556
CdTV-To[MX;SinB52;83].B.AF226666
CdTV-To[MX;Cin].B.U57458
CdTV-To[MX;SinIC;83].B.AF101478
SiGMCRV-[CR].B.X99551
SiGMHNV-[HN].B.Y11098
ToLCSinV-[NI;SL].B.AJ508783
BDMV-[CO;87].B.M88180
SiGMV-[US;Flo].B.AF039841
AbMV-[US;HW].B.U51138
AbMV-[DE].B.X15984
SiYMYuV-[MX;Yuc;05].B.DQ875873
CoYSV-[MX;Yuc;05].B.DQ875869
ToMoV-[US;Flo;89].B.L14461
ToMoV-[PR;04].B.AY965901
ToMoTV-[CU].B.AF012301
ToMHV-[CU;Qui].B.Y14875
PYMV-To[GP;Tom].B.AY120883
PYMV-To[PR;Tom;04].B.AY965898
PYMTV-[TT;Tom].B.AF039032
PYMV-Po[VE].B.D00941
PYMPV-[PA;Div;Tom].B.Y15033
DesLDV-[MX;Yuc;05].B.DQ875871
CLCrV-AZ[US;Cal;91].B.AY742221
CLCrV-AZ[MX;Son;91].B.AF480941
SLCV-[US;Imp;79].B.M38182
SMLCV-[US;Imp;79].B.AF421553
MCLCuV-CR[CR;Gua;98].B.AF440790
CuLCrV-[US;Ari;91].B.AF327559
CuLCrV-[US;Cal;98].B.AF224761
BCaMV-[MX;Son;86].B.AF110190
EuMV-[MX;Yuc;04].B.DQ318938
CYVV-[VN;Hoa;00].B.AY727904
CoGMV-[NV;Han;05].B.DQ641688
ToLCNDV-IN[IN;ND;05].B.DQ169057
ToLCNDV-[IN;Luf;05].Bt.AY939924
ToLCNDV-IN[IN;ND;Svr;92].B.U15017
ToLCNdV-[PkT5].Bseq.AY150305
ToLCNDV-IN[IN;Mee;Pot2;02].B.AY158080
ToLCNDV-IN[IN;Hap;Pot;05].B.EF043233
ToLCNDV-IN[IN;Me;Pot;05].B.EF043232
ToLCNdV-[PkT1].Bseq.AY150304
ToLCNdV-[PkSn].Bseq.AJ620188
ToLCNDV-[PK;Chi;04].B.DQ116882
ToLCNDV-[PK;Lah;04].B.AM392426
ToLCGV-[IN;Var;01].B.AY190291
ToLCNDV-Svr[BG;Jes;05].B.AJ875158
ToLCNDV-IN[IN;Luc].B.X89653
ToLCNDV-IN[IN;ND;AVT1].B.AY438563
SLCCNV-CN[VN;B].B.AF509742
SLCCNV-CN[CN;Hn61;05].B.AM260207
SLCCNV-IN[IN;Coi;Pum].B.AY184488
SLCPHV-PH[PH;Mun].B.AB085794
LYMV-[VN].B.AF509740
SLCMV-IN[IN;Adi].B.AJ579308
SLCMV-IN[IN;Kat].B.AJ575821
SLCMV-LK[LK;Col;98].B.AF314738
ICMV-Ker[IN;Ker;02].B.AJ512823
ICMV-IN[IN;Mah;88].B.AJ314740
ICMV-IN[IN;Tri;86].B.Z24759
ICMV-IN[IN;Mah2;88].B.AY730036
TYLCThV-YY[TH;ChMay].B.AY514633
TYLCThV-ZZ[TH;SaNa].B.AY514635
TYLCThV-YY[TH;NoK].B.AY514634
TYLCTHV-XX[TH;1].B.X63016
TYLCTHV-XX[TH;2].B.AF141897
TYLCKaV-[TH;Kan2;Egg;05].B.AF511528
TYLCKaV-[TH;Kan;01].B.AF511527
TYLCKaV-[VN;05].B.DQ169055
PepYLCIV-YY[ID;05].B.AB267835
PepYLCIV-XX[ID;Age;05].B.AB267839
PepYLCIV-XX[ID;Tom;05].B.AB267837
ClGMV-[VN;Son;05].B.DQ641693
48.0
45
40
35
30
25
20
15
10
5
0
